# Growth Rates, Carcass Traits, Meat Yield, and Fatty Acid Composition in Growing Lambs under Different Feeding Regimes

**DOI:** 10.3390/life13020409

**Published:** 2023-02-01

**Authors:** Faisal A. Alshamiry, Abdulrahman S. Alharthi, Hani H. Al-Baadani, Riyadh S. Aljumaah, Ibrahim A. Alhidary

**Affiliations:** Department of Animal Production, College of Food and Agriculture Science, King Saud University, P.O. Box 2460, Riyadh 11451, Saudi Arabia

**Keywords:** carcass traits, feeding regimes, fatty acids, growing lamb, meat quality

## Abstract

A total of 75 male Awassi (mean BW 23.5 ± 2.0 kg; 3 months old) were used in an 84-day trial to investigate the effects of different feeding regimes on productive performance, carcass characteristics, and meat quality, and the fatty acid profile of growing lambs. Animals were randomly allocated into 3 groups of 25 lambs each. The dietary treatments were as follows: (1) whole barley grain (60%) plus alfalfa hay (40%; GB-AH; the basal diet); (2) a concentrate pelleted diet plus alfalfa hay (CP-AH); and (3) a complete pelleted diet (CPD). Feed intake was measured weekly, and all lambs were weighed every two weeks for an evaluation of the productive parameters. Blood samples were collected from all lambs for the measurement of biochemical and enzymatic variables. At the end of the experiment, 13 lambs from each treatment were slaughtered to evaluate the carcass characteristics, meat quality, and fatty acid composition. The final body weight, body weight gain, average daily gain, and feed efficiency of lambs were lowest (*p* < 0.05) in lambs on the grain and alfalfa diet compared with the other groups. Feeding lambs either the CP-AH or CPD diets resulted (*p* < 0.05) in increases in slaughter weight, carcass weights (hot and cold), the percentage of liver and shoulder, carcass length, back fat thickness, and the area of longissimus thoracis muscle compared with those lambs on the GB-AF diet. The proportion of saturated fatty acids in meat was greater (*p* = 0.04) in lambs fed on the GA-AH diet than in those of lambs fed on the pelleted diets. Lambs on the CP-AH diet had (*p* < 0.05) the highest ratios of PUFA to SFA and omega 6 to omega 3, and the proportion of omega 6. The atherogenic and thrombogenic indexes were lower (*p* < 0.05) in the CP-AH group compared with the GB-AH group. In conclusion, the results indicate that feeding growing lambs on concentrate pellets instead of whole barley grain improves the growth rate, traits, meat quality, and fatty acid profile, which have important implications for productivity, efficiency, and profitability in the livestock industry.

## 1. Introduction

Feeding livestock animals plays a crucial role in animal production, and there is growing concern regarding feed raw materials (availabilities and costs) and their direct impacts on livestock productivity and efficiency, which can substantially contribute to the profitability of the livestock industry [1,2,3,4]. Feedstuffs and nutrition play an important role in the livestock production chain (e.g., a link between crop yield, animal products, and the processing). A higher demand and competition regarding supplies result in stressed market conditions in which feedstuff operators and producers need to continuously balance activities, considering animal performance and consumer demand. Since animal feed contributes to up to 70% of the total operating productivity costs in animal production, and the profits in the chain are dynamic and usually under pressure, improvements in feed quality and feeding management have received much attention in order to optimize productivity and efficiency [4,5,6,7].

In arid and semi-arid regions, sheep are raised under harsh conditions in terms of shortages in natural resources, lower forage availability, higher feed, and energy costs. Under these conditions, three main feeding systems are used for feeding livestock: cereals plus forages (mainly barley and alfalfa hay), concentrate pellets plus forages, or complete pelleted diets [7,8]. Moreover, the quantity and quality of feeds are mainly dependent on the type, proportion, and availability of the feedstuffs that are used, as well as the feed processing, all of which can directly affect the changes in growth rate, carcass yield, meat quality, and composition [4,9,10,11]. Therefore, the objectives of the study were to investigate the effects of different feeding regimes on productive performance, carcass characteristics, meat quality, and fatty acid contents in growing lambs.

## 2. Materials and Methods

### 2.1. Animal Welfare and Ethics Clearance

The research experiment was carried out at King Saud University in Riyadh, Saudi Arabia. All animals and procedures used in this experiment followed to the Animal Welfare Act of Practice for the Care and Use of Animals for Scientific Purposes, with approval from the King Saud University Research Ethics Committee (REC-KSU; Ethics Reference No: KSU-SE-22-26).

### 2.2. Animals and Management Practices

A total of 75 growing Awassi lambs with average initial body weights of 23.5 ± 2.0 kg, ages of 3 months, and a good healt condition were used in an 84-day trial. The lambs were purchased from a local livestock market 14 days before the beginning of the experiment, and then they were transported using an animal transport truck to the Experimental Station of Animal Production Department, King Saud University. On the day of arrival, the animals were immediately ear tagged, weighed, immunized against comment infections, and treated for both internal and external parasites of sheep. Thereafter, lambs were allocated randomly into 15 replicates of 5 lambs each and housed in shaded group pens (4.0 m long by 3.0 m wide); each pen was provided with a feed trough and water bucket. Lambs were given a two-week adaptation period in these pens. On day 1, lambs were randomly assigned to 1 of 3 dietary treatments (5 groups in each treatment), which were as follows: (1) whole barley grain (60%) plus alfalfa hay (40%; GB-AH; the basal diet); (2) a concentrate pelleted diet plus alfalfa hay (CP-AH); and (3) a complete pelleted diet (CPD). All three feeds were formulated (Table 1) in order to meet the nutritional requirements for growing lambs according to NRC [12]. 

### 2.3. Feed Analyses 

Samples of feed were collecetd frequently every month from each treatment and then pooled (5%) at the end of the study for nutrient composition determination. Feed samples were dried in an oven at 100 °C for 4 h for determination of dry matter (DM) and then incinerated in a muffle furnace at 550 °C for 3 h in order to determine ash content. The crude protein (CD) contained in each sample was measured by using an elemental analyzer. The samples containing neutral and acid detergent fibers (NDF and ADF, respectively) were measured following the methodologies described by Van Soest et al. [13] and the AOAC [14], respectively. The fatty acids compositions in feed samples were determined according to the methods described by Barros et al. [15].

### 2.4. Growth Rate and Feed Efficiency

All lambs were weighted, using an electronic small-animal scale, on day 1, and then every 2 weeks, to calculate the initial body weight, changes in body weight, and average daily gain. Feed intake for each pen was recorded weekly by measuring the difference between feed offered and feed refused. The feed conversion ratio (FCR) was calculated by dividing the amount of daily intake on average daily gain, and then expressed as kg of DMI to kg BW. 

### 2.5. Blood Sample Processing and Analysis

On days 1, 40, and 84, blood samples (10 mL) were taken from all lambs through jugular venipuncture. Aliquots of 10 mL of blood were placed in 2 Vacutainer tubes devoid of additives in order to obtain serum. The serum concentrations of glucose, non-esterified fatty acid (NEFA), total protein, albumin, urea, creatinine, total cholesterol, high-density lipoprotein (HDL), low-density lipoprotein (LDL), triglyceride (TG), creatine kinase (CK), aspartate aminotransferase (AST), alanine aminotransferase (ALT), and β-hydroxybutyrate were determined according to the manufacturer’s instructions. Commercial kits (Randox Laboratories, Antrim, United Kingdom) and a microplate reader (Multiskan EX, Thermo Fisher Scientific Inc., Waltham, MA, USA) were used for analysis.

### 2.6. Carcass Characteristics and Meat Quality

On day 84, lambs (13 animals per treatment) were firstly deprived of the diets for 16 h and then slaughtered following the Islamic slaughtering method. During the slaughtering processes, the live body weight, empty and hot carcass, organs (including heart, liver, stomach, kidneys, and tail) were weighted and then stored at 4 °C. After 24 h of slaughter, the carcasses were weighed again to determine cold carcass weight, dressing percentage, and chilling losses for each carcass. Thereafter, the 6th–13th ribs were separated (right side). Then, pH values of meat were determined by using a pH meter. The back fat thickness above the 6th and 10th ribs was recorded by using a digital ruler. The 12th rib from each carcass was used to measure the longissimus thoracis muscle and to determine the meat color value by using a Minolta Chroma Meter (Konica Minolta, CR-400-Japan) with a CIELAB Color System for the color values (L * = lightness; a * = redness; and b * = yellowness). An approximately 300 g sample of muscle (2.5 cm thick) was used to determine the myofibril fragmentation index (MFI) [16], water-holding capacity (WHC) [17], and cooking loss (CL) [18]. A texture analyzer (TA. HD, Stable Micro Systems, Surrey, UK), with a compression-plate attachment was used to determine the meat chewiness, hardness, springiness, and cohesiveness. 

### 2.7. Fatty Acid Analysis

Using the procedure described by Barros et al. [15], samples of subcutaneous fat were obtained from the ribs and longissimus thoracis muscle of each carcass to evaluate fatty acid contents. In summary, 3 gm of dry meat was thoroughly homogenized in a 60 mL mixture of chloroform and methanol (2:1), followed by filtering by (#4 filter paper, >20 to 25 μm) and an addition of 12 mL of a 0.88% KCl solution. This was centrifuged at 2000× *g* for 30 min at room temperature before being transferred to a reaction vial and nitrogen-dried. The dry samples received 0.5 mL of (0.5 N) NaOH in methanol, which was then heated in a heating block for 5 min at 100 °C before being cooled to room temperature. Total fat (g/100 g muscle) was calculated at the end of this process. The extract received 0.5 mL of boron trifluoride in methanol. The extracts were then heated for 5 min at 100 °C before being cooled to room temperature. A total of 0.5 mL of hexane and 1 mL of saturated NaCl were added, and the mixture was centrifuged at 1500× *g* for 5 min. The methyl esters of fatty acids found in the upper phase were isolated. The capillary column SP-2340 was then fused in silica using gas chromatography–mass spectrometry (GC–MS) with a flame-ionization detector under the following conditions: initial temperature, 150 °C; initial isotherm, 5 min; the temperature was increased 1 °C per minute up to 160 °C; intermediate isotherm, 11 min, followed by a temperature increase of 7 °C per minute up to 230 °C; final isotherm, 9 min; injector temperature, 200 °C; and detector temperature, 250 °C. The carrier gas was helium (1.5 mL/min). A combination of standard fatty acids was used to identify the fatty-acid peaks (Larodan Fine Chemicals AB). Fatty acids (FAs) were computed and calculated as a proportion of identified fatty-acid methyl esters using chromatogram peak regions.

### 2.8. Statistical Analyses

All data were analyzed by using a complete randomized design with general linear model procedures of statistical analysis software (SAS Institute Inc., Cary, NC, USA). The statistical model included the type of diet (feeding regime), collection day and animal within treatment, carcass and meat quality characteristics, and fatty acid composition data for the GLM model of SAS. Data are reported with the least square mean (±SE), and the differences between them are significantly considered at *p* < 0.05.

## 3. Results

### 3.1. Dry Matter Intake (DMI), Growth Rate, and Feed Efficiency

The effects of feeding regimes on the productive performance of growing lambs are presented in Table 2. The growth rate, feed intake, and feed conversion rate were affected (*p* < 0.05) by dietary treatment. Feeding lambs on the pelleted diets (CP-AH or CPD treatment) showed increases in final BW (*p* = 0.03), BWG, relative growth (*p* = 0.02), and ADG (*p* = 0.01), as well as (*p* = 0.03) improvements in FCR when compared with lambs fed barley plus alfalfa hay (GB-AH group). DMI was greater (*p* = 0.02) in lambs in the CP-AH treatment group than in lambs in the CPD treatment group; the GB-AH group had an intermediate DMI, which was not significantly different (*p* > 0.05) compared with other dietary groups. 

### 3.2. Serum Metabolic Profile

Differences among treatments regarding the concentration of the serum biochemical and enzymatic variable measured in the study are presented in Table 3. Different feeding regimes had no effect (*p* > 0.05) on the measured biochemical or enzymatic variables that were compared, with the exception of serum concentrations of glucose, urea, HDL, and LDL (*p* < 0.05). Lambs fed the CP-AH diet had the greatest mean serum concentrations of glucose (*p* = 0.01; 4.45 mM) and urea (*p* = 0.04; 2.52. mM) compared with lambs in the other treatments. Lambs fed the CPD diet showed increased serum concentrations of HDL (*p* = 0.03) compared with lambs fed on the CP-AH diet. 

### 3.3. Carcass Traits and Meat Characteristics

Differences among feeding regimes regarding carcass and meat quality characteristics are presented in Table 4 and Table 5, respectively. The percentages of dressing and chilling losses, organs (kidneys and heart) in carcass, the widths of carcass and rump, leg length, fat wall thickness, pH and color values of meat, and meat composition, as well as the percentages of WHC and cooking loss, shear force, springiness, cohesiveness and chewiness, did not differ (*p* > 0.05) among dietary treatment. In comparison with lambs in the GB-AH group, feeding lambs with either the CP-AH or CPD diets resulted (*p* < 0.05) in increases in slaughter weight, carcass weights (hot and cold), the percentage of liver and shoulder, lengths of internal and external carcass and back fat thickness, and the area of longissimus thoracis muscle. Lambs on the GB-AH diet had the highest (*p* < 0.05) percentages of tail, rack, loin, FSH + breast, and total visceral depot fat, as well as the highest MFI and hardness, when compared with lambs of other treatments. 

### 3.4. Fatty Acid Classes and Indices

The differences among dietary treatments in terms of fatty acid composition and lipid nutritional indices in the longissimus thoracis muscle of growing lambs are presented in Table 6. The major three saturated fatty acids were palmitic (C16:0; 22.6%), stearic (C18:0; 17.4%), and tetradecanoic (C14:0; 3.11%) acids. The proportions of C18:0 and C14:0 were the greatest (*p* < 0.05) for lambs fed on barley and alfalfa hay (GB-AH group). Consequently, the proportion of saturated fatty acids was greater (*p* = 0.04) in lambs fed on the GA-AH diet than in those of lambs fed on the pelleted diets (49.15 vs. 46.73%). Oleic acid (C18:1 ω 9; 36.3%) and linoleic acid (C18: 2 ω 6) were the major unsaturated fatty acids (MUFA and PUFA), and the proportions of these two fatty acids were greatest (*p* < 0.05) in lambs fed on the CP-AH diet. Feeding lambs on the pelleted diets resulted in increases in the proportions of MUFA and PUFA (*p* = 0.03 and 0.02, respectively). Regarding the lipid nutritional indices, feeding lambs pelleted concentrate and alfalfa hay (CP-AH diet) resulted in increases in the PUFA-to-SFA (0.16) and omega-6-to-omega-3 (5.93) ratios and the proportion of omega 6 (5.87%), and also caused reductions in the atherogenic index (0.63) and thrombogenic index (1.48).

## 4. Discussion

In the current study, providing alfalfa hay with concentrate pellets increased feed intake by 14.2% compared with the total mixed ratio diet in pellet diets. The addition of roughage to concentrate pellets improves rumen fermentation and feed intake in fattening animals [6,19]. The consumption of roughages with concentrate pellets can encourage rumen motility, increase size and muscular development, and promote rumination [20,21]. It is generally accepted that an increased fiber intake results in the physical filling of the rumen and, thus, regulates intake [22,23,24,25]. 

Previous studies on productive performance in lambs have indicated that growth rate is increased by feeding ruminant animals total mixed ration diets in a pellet form, rather than concentrate or complete feeds [26,27,28,29]. This beneficial effect on growth rate is supported by the results of Atti and Mahouachi [30], who observed an 18.3% increase in body-weight gains in lambs fed high-concentrate pellets compared with that of lambs fed grain-feeding pastures. Similarly, feeding growing lambs pellet diets in the current study resulted in increases in body weight gain (24.2%; 20.25 vs. 16.3 kg) and average daily gain (23.3%; 240.5 vs. 195.0 g/d) compared with lambs fed barley grain and alfalfa hay. The mechanism by which a concentrate pellet diet improves growth rate is mainly increasing the daily feed intake or improving feed efficiency regarding the majority of animals’ nutritional requirements, particularly the micronutrients, which, in turn, have a positive effect on feed efficiency and growth rate. 

Although all serum concentrations of biochemical and enzymatic parameters were within the reference ranges of serum metabolic concentrations for sheep reported by Aiello [31], increases were observed in the glucose and urea concentrations in serum in lambs fed concentrate pellet and alfalfa hay in the current study. There is evidence of alterations in the extent and pathways of utilization for these processed forages [32,33]. These increases in circulating glucose and urea to the rumen as metabolic pathways (protein and carbohydrate digestibility) can be caused by feeding livestock animals a long alfalfa hay [27,34]. 

Evidence from previous studies indicates that the quality and quantity of the feeding system can directly and indirectly affect the carcass characteristics and meat quality of ruminants, causing significant improvements by increasing growth rate, providing the nutritional requirements, quickly achieving the target weights, and causing alterations in enzymatic and hormonal secretions, or improvements in immune and health status [1,4,19,35]. Feeding lambs concentrate pellets increased the carcass variables (including the slaughter weights, weights of wholesale cuts and organs, and linear measurements of carcass and fatness scores) when compared with lambs fed grains [1,27,28,36]. Similarly, lambs fed pellet diets (either concentrate or complete pellets) in the current study showed increases in slaughter weight (9.4%), hot carcass weight (11.1%), shoulder (10.8%), backfat thickness (16.9%), and the area of longissimus thoracis muscle (16.8%), all of which can be considered as improvements in feed efficiency and growth rate.

Meat quality is a multidimensional concept encompassing organoleptic, nutritional, and microbiological characteristics, which, in turn, can be regulated by a variety of factors that are intrinsic and extrinsic to ruminant animals. Fatty acids content has a vital role in the nutritional value and organoleptic characteristics of meat [37]. Numerous meat science studies have observed that alterations in fatty acid profile in the meat of livestock animals mainly depend on the type of feed and ingredients used [4,6,30,36]. For instance, feeding fattening lambs concentrate pellets instead of barley resulted in an increase from 35.0% to 35.9% in the MUFA proportion in the longissimus thoracis muscle [28]. In addition, the main fatty acids in the longissimus thoracis muscle of lambs were oleic, palmitic, and stearic acids [28,38,39,40]. 

Despite the ω-6/ω-3 ratio being only one factor of the pathogenesis of cardiovascular and other diseases, its lower value seems to decrease the risk of chronic diseases [41,42]. These results are consistent with the results obtained by the current study that feeding lambs concentrate diets instead of whole cereals causes an increase in unsaturated fatty acids (MUFA and PUFA), and reductions in SFA, AI, and TI, all of which have a positive effect for meat consumers.

## 5. Conclusions

Under the conditions of this study, the results indicate that feeding growing lambs concentrate pellets instead of whole barley grain improved their growth rate, traits, meat quality, and fatty acid profile, which have important implications for productivity, efficiency, and profitability in the livestock industry. In addition, these results can have beneficial contributions to human health. Therefore, it is better for lamb producers to use concentrate pellets in terms of productive quantities and qualities as well as economic efficiency. 

## Figures and Tables

**Table 1:** Ingredients and chemical composition of the experimental diets ^1^.

Item, Unit	Dietary Treatments ^1^		
	GB-AH	CP-AH	CPD
Ingredients, % of dietary dry matter			
Barley grain	60.00	0.00	0.00
Alfalfa hay, 5 cm	40.00	40.00	0.00
Yellow corn	0.00	27.00	35.14
Grand Alfalfa hay	0.00	0.00	22.13
Wheat bran	0.00	15.00	18.00
Soybean meal	0.00	13.25	9.80
Wheat straw	0.00	0.00	7.00
Molasses	0.00	0.90	1.60
Limestone	0.00	1.25	3.00
Dicalcium phosphate	0.00	1.20	1.40
Acid buffer	0.00	0.66	0.80
Salt	0.00	0.50	0.83
Vit min premix ^2^	0.00	0.24	0.30
Nutrient composition, dry matter basis			
Dry matter, (%)	90.48	89.11	89.91
Ash, %	6.38	7.17	7.23
Crude protein, %	14.03	14.66	14.34
Ether extract, %	1.34	1.89	1.64
Neutral detergent fiber, %	36.01	34.67	30.34
Acid detergent fiber, %	17.26	15.81	11.78
Metabolizable energy, Mcal/kg	2.89	2.93	2.85
Lipid profile, %			
Saturated fatty acids	28.63	29.40	32.06
Monounsaturated fatty acids	12.94	34.11	23.53
Polyunsaturated fatty acids	58.42	36.50	44.41

^1^ GB-AH = 60% of grain barley plus 40% of alfalfa hay; CP-AH = concentrate pelleted diet plus alfalfa hay; and CPD = complete pelleted diet. ^2^ Contained per kg: 10,000 IU vitamin A, 1000 IU vitamin D, 20 IU vitamin E, 300 mg Mg, 24 mg Cu, 0.6 mg Co, 1.2 mg I, 60 mg Mn, 0.3 mg Se, 60 mg Zn.

**Table 2:** Means of body weight (BW), body weight gain (BWG), average daily gain (ADG), dry matter intake (DMI), and feed conversion ratio (FCR) of growing lambs fed with different feeding systems.

Item, Unit	Dietary Treatments ^1^			SE.	*p* Value
	GB-AH	CP-AH	CPD		
Initial BW, kg	23.8	23.6	23.6	0.22	0.94
Final BW, kg	40.2 ^b^	44.6 ^a^	43.2 ^a^	2.78	0.03
BWG, kg	16.3 ^b^	20.9 ^a^	19.6 ^a^	2.43	0.02
Relative growth, %	51.25 ^b^	61.58 ^a^	58.68 ^a^	5.67	0.02
ADG, g/d	195 ^b^	248 ^a^	233 ^a^	25.56	0.01
DMI, kg/d	1.44 ^ab^	1.53 ^a^	1.34 ^b^	0.12	0.02
FCR, kg DM: kg BW	7.44 ^a^	6.21 ^b^	5.73 ^b^	0.63	0.03

^a,b^ Means without a common superscript differ within a row (*p* < 0.05). ^1^ Values for growing lambs (n = 75); GB-AH = 60% of grain barley plus 40% of alfalfa hay; CP-AH = concentrate pelleted diet plus alfalfa hay; CPD = complete pelleted diet.

**Table 3:** Effects of different feeding systems on the serum concentrations of biochemical and enzymatic variables in growing lambs.

Analyte, Unit ^2^	Dietary Treatments ^1^			SE.	*p* Value
	GB-AH	CP-AH	CPD		
Glucose, mM	4.15 ^b^	4.45 ^a^	4.18 ^b^	0.16	0.01
NEFA, mM	0.27	0.26	0.25	0.03	0.67
Total protein, g/L	56.13	56.79	57.25	3.35	0.25
Albumin, g/L	31.79	33.90	33.25	2.11	0.12
Urea, mM	2.30 ^b^	2.52 ^a^	2.37 ^b^	0.12	0.04
Creatinine, µM	99.2	94.9	95.93	6.67	0.18
Total cholesterol, mM	2.42	2.39	2.34	0.09	0.21
HDL, mM	1.10 ^a^	1.01 ^b^	1.13 ^a^	0.04	0.03
LDL, mM	0.86 ^ab^	0.93 ^a^	0.77 ^b^	0.13	0.02
Triglyceride, mM	1.01	0.99	0.97	0.08	0.34
AST, U/L	87.8	93.7	87.9	7.04	0.09
ALT, U/L	15.2	16.4	16.5	1.43	0.46
CK, U/L	225	241	238	18.11	0.23
*β*-Hydroxybutyrate, mM	9.09	9.13	8.93	0.67	0.56

^a,b^ Means without a common superscript differ within a row (*p* < 0.05). ^1^ Values for growing lambs (n = 75); GB-AH = 60% of grain barley plus 40% of alfalfa hay; CP-AH = concentrate pelleted diet plus alfalfa hay; CPD = complete pelleted diet. ^2^ ALT = alanine aminotransferase; AST = aspartate aminotransferase; CK = creatine kinase; HDL = high-density lipoprotein; LDL = low-density lipoprotein; NEFA = non-esterified fatty acids.

**Table 4:** Effects of different feeding systems on the carcass characteristics of growing lambs.

Item, Unit ^2^	Dietary Treatments ^1^			SE.	*p* Value
	GB-AH	CP-AH	CPD		
Carcass profile					
Slaughter BW, kg	40.21 ^b^	44.62 ^a^	43.33 ^a^	1.12	0.02
Empty BW, kg	34.37 ^b^	39.50 ^a^	37.79 ^a^	1.87	0.02
Hot carcass, kg	18.91 ^b^	21.43 ^a^	20.58 ^a^	1.34	0.04
Cold carcass, kg	18.38 ^b^	20.83 ^a^	19.97 ^ab^	1.09	0.05
Dressing, %	46.33	48.00	47.50	2.23	0.19
Chilling losses, %	2.57	2.81	2.94	0.46	0.34
CCI, kg/cm	0.29	0.27	0.25	0.07	0.25
Organs weight, %					
Liver	1.74 ^b^	1.86 ^a^	1.71 ^b^	0.06	0.03
Kidneys	0.27	0.29	0.31	0.05	0.11
Heart	0.34	0.35	0.32	0.03	0.23
Empty stomach	2.95 ^b^	2.87 ^b^	3.55 ^a^	0.15	0.04
Empty intestine	2.72 ^b^	2.92 ^ab^	3.11 ^a^	0.21	0.03
Tail	5.60 ^a^	5.43 ^a^	4.92 ^b^	0.27	0.05
Wholesale cuts, %					
Shoulder	28.62 ^b^	32.02 ^a^	31.41 ^a^	1.56	0.04
Rack	8.02 ^a^	7.37 ^b^	7.41 ^b^	0.34	0.01
Loin	13.56 ^a^	13.19 ^b^	13.04 ^b^	0.28	0.03
Leg	33.31	33.52	33.53	0.31	0.43
FSH + Breast	16.49 ^a^	13.89 ^b^	14.61 ^ab^	1.78	0.02
Carcass linear measurement, cm					
Internal carcass length	66.88 ^b^	74.13 ^a^	73.27 ^a^	3.56	0.03
External carcass length	67.00 ^b^	71.31 ^a^	70.77 ^a^	2.25	0.03
Carcass width	31.75	33.31	32.83	2.12	0.09
Rump width	41.06	40.13	40.15	1.43	0.37
Leg length	42.00	43.63	42.94	1.23	0.78

^a,b^ means without a common superscript differ within a row (*p* < 0.05). ^1^ Values for growing lambs (n = 39); GB-AH = 60% of grain barley plus 40% of alfalfa hay; CP-AH = concentrate pelleted diet plus alfalfa hay; CPD = complete pelleted diet. ^2^ CCI = Carcass Compactness Index; FSH + Breast = foreshank and hind hank + breast.

**Table 5:** Effects of different feeding systems on meat quality of growing lambs.

Item, Unit ^2^	Dietary Treatments ^1^			SE.	*p* Value
	GB-AH	CP-AH	CPD		
Meat quality					
Back fat, mm	3.35 ^b^	3.78 ^ab^	4.05 ^a^	0.42	0.04
Body wall fat, mm	6.05	5.32	6.38	0.31	0.18
Area 12th rib, cm^2^	15.56 ^b^	18.63 ^a^	17.77 ^a^	1.12	0.03
pH value	5.75	5.73	5.81	0.18	0.56
Color components					
L *	37.23	37.54	39.74	3.34	0.34
a *	14.76	14.41	15.03	1.07	0.23
b *	10.98	10.92	9.91	1.23	0.78
Visceral depot fat,%					
Mesentery fat	0.85 ^a^	0.76 ^b^	0.82 ^a^	0.04	0.03
Omental fat	1.17 ^a^	0.67 ^b^	0.76 ^b^	0.23	0.02
Pericardial fat	0.18	0.19	0.18	0.02	0.54
KKCF	0.70 ^a^	0.47 ^b^	0.40 ^b^	0.13	0.04
Total	2.89 ^a^	2.10 ^b^	2.16 ^b^	0.21	0.03
Meat composition, %					
Meat	25.28	22.75	24.6	3.21	0.06
Bone	46.50	47.39	47.18	1.56	0.18
Fat	23.85	24.52	24.04	0.78	0.21
Trimmings	4.37	5.34	4.18	0.83	0.11
Texture profile analysis					
WHC	34.51	32.80	33.91	1.43	0.21
Cooking Loss, %	34.61	33.80	35.76	1.67	0.09
MFI	50.20 ^a^	46.05 ^b^	41.74 ^c^	3.22	0.04
Shear force, N	12.78	11.70	13.06	1.78	0.07
Hardness, N	10.14 ^a^	9.10 ^b^	10.96 ^a^	1.03	0.04
Springiness	0.69	0.70	0.68	1.06	0.46
Cohesiveness	0.55	0.57	0.53	0.06	0.78
Chewiness	4.00	3.97	4.14	0.21	0.54

^a–c^ means without a common superscript differ within a row (*p* < 0.05). ^1^ Values for growing lambs (n = 39); GB-AH = 60% of grain barley plus 40% of alfalfa hay; CP-AH = concentrate pelleted diet plus alfalfa hay; CPD = complete pelleted diet. ^2^ L * = lightness; * = redness; b * = yellowness; KKCF = kidney knobs channel fat; MFI = myofibril fragmentation index; WHC = water-holding capacity.

**Table 6:** Means of fatty acid composition (%) and lipid nutritional indices of m. longissimus dorsi from growing lambs on different feeding regimes.

Item, Unit ^2^	Dietary Treatments ^1^			SE.	*p* Value
	GB-AH	CP-AH	CPD		
C10:0	0.18 ^b^	0.20 ^b^	0.35 ^a^	0.03	0.01
C12:0	0.29	0.35	0.30	0.07	0.56
C13:0	ND	0.18	0.17	ND	ND
C14:0	3.44 ^a^	2.60 ^b^	3.30 ^a^	0.65	0.01
C15:0	1.07 ^a^	0.79 ^b^	0.78 ^b^	0.21	0.02
C16:0	22.86	22.11	22.99	1.43	0.23
C17:0	3.01 ^a^	2.58 ^b^	2.39 ^b^	0.26	0.04
C18:0	18.15 ^a^	17.96 ^a^	16.04 ^b^	1.07	0.02
C19:	0.15 ^b^	0.19 ^a^	0.18 ^a^	0.02	0.03
SFA	49.15 ^a^	46.97 ^b^	46.49 ^b^	2.11	0.04
C14:1 ω5	0.24 ^b^	0.34 ^a^	0.27 ^b^	0.07	0.05
C14:1 ω7	0.24 ^b^	0.40 ^a^	0.38 ^a^	0.08	0.02
C16:1 ω5	0.36 ^b^	0.31 ^b^	0.50 ^a^	0.11	0.02
C16:1 ω7	2.56 ^b^	2.26 ^b^	3.13 ^a^	0.34	0.03
C18:1 ω 5	1.12 ^a^	1.05 ^ab^	1.02 ^b^	0.09	0.05
C18:1 ω 7	3.60 ^b^	3.85 ^ab^	4.02 ^a^	0.32	0.05
C18:1 ω 9	36.48 ^ab^	37.13 ^a^	35.26 ^b^	1.56	0.02
C20:1 ω 9	0.19	ND	0.43	ND	ND
C22:1 ω 11	ND	ND	0.41	ND	ND
MUFA	44.79 ^b^	45.35 ^a^	45.41 ^a^	0.48	0.03
C16:3 ω 4	1.13 ^b^	0.82 ^b^	2.05 ^a^	0.54	0.01
C18:2 ω 6	3.54 ^b^	5.32 ^a^	4.35 ^ab^	1.34	0.02
C18:3 ω 3	0.41	0.48	0.50	0.12	0.07
C18:4 ω 3	0.56	0.51	0.51	0.08	0.12
C18:3 ω 6	ND	0.15	0.15	ND	ND
C20:4 ω 6	0.43 ^b^	0.39 ^b^	0.54 ^a^	0.06	0.03
PUFA	6.06 ^b^	7.68 ^ab^	8.10 ^a^	1.63	0.02
Lipid nutritional indices					
PUFA/SFA	0.12 ^b^	0.16 ^a^	0.17 ^a^	0.02	0.01
n-6	3.97 ^b^	5.87 ^a^	5.04 ^a^	0.89	0.02
n-3	0.97	0.99	1.00	0.05	0.56
n-6/n-3	4.09 ^c^	5.93 ^a^	5.02 ^b^	0.86	0.01
AI	0.74 ^a^	0.63 ^b^	0.71 ^a^	0.08	0.02
TI	1.61 ^a^	1.48 ^b^	1.49 ^b^	0.09	0.03

^a–c^, means without a common superscript differ within a row (*p* < 0.05). ^1^ Values for growing lambs (n = 39); GB-AH = 60% of grain barley plus 40% of alfalfa hay; CP-AH = concentrate pelleted diet plus alfalfa hay; and CPD = complete pelleted diet. ^2^ Al = atherogenic index; MUFA: monounsaturated fatty acids; n-3 = omega 3; n-6 = omega 6; ND: PUFA: = not detected; polyunsaturated fatty acids; SFA: saturated fatty acids; and TI = thrombogenic index.

## Data Availability

All data collected and analyzed during this study are available from the corresponding author on request.
